# Absence of some common organ-specific and non-organ-specific autoimmunity in autoimmune polyendocrinopathy candidiasis ectodermal dystrophy

**DOI:** 10.1530/EC-12-0074

**Published:** 2013-02-25

**Authors:** Nicolas Kluger, Kai Krohn, Annamari Ranki

**Affiliations:** Department of Dermatology, Allergology and Venereology Institute of Clinical Medicine, Skin and Allergies Hospital, University of Helsinki, Helsinki University Central Hospital Meilahdentie 2, PO Box 16000029, Helsinki Finland; 1 Clinical Research Institute HUCH Ltd., Biomedicum Helsinki 1 Haartmaninkatu 8, PO Box 70000290, HelsinkiFinland

**Keywords:** APECED, AIRE, antinuclear antibodies, anti-desmoglein antibodies, HLA, MHC

## Abstract

**Background:**

Autoimmune polyendocrinopathy candidiasis ectodermal dystrophy (APECED) is a rare autosomal recessive disorder caused by mutations of the autoimmune regulator (*AIRE*) gene, whose loss of function leads to the escape of self-reactive T cells from the thymus and autoimmunity. APECED patients typically develop tissue-specific autoantibodies and anti-cytokine antibodies. Consequently, various endocrine and non-endocrine autoimmune disorders appear. However, only a certain number of autoimmune diseases develop, while some common autoimmune conditions have not been reported or are seen only anecdotally.

**Objective:**

We investigated the clinical manifestations and occurrence of antinuclear antibodies (AN-Abs) and antibodies against extractable nuclear antigens, citrullinated peptide, and transglutaminase in 24 patients and against bullous pemphigoid antigen 180 and desmogleins 1 (Dsg1) and Dsg3 in 30 patients of a Finnish cohort of APECED patients.

**Results:**

Despite the loss of central tolerance, the autoantibodies investigated were not overrepresented among the APECED patients. None of the patients had a history of autoimmune connective tissue disease, rheumatoid arthritis, celiac disease, or autoimmune cutaneous bullous disorders. Altogether, 25% (6/24) had low-titer (1:80) AN-Abs. Two patients had anti-BP180 antibodies and two others had anti-Dsg3 antibodies without any cutaneous or mucosal symptoms. No anti-citrullinated peptide and anti-transglutaminase reactivity was found.

**Conclusions:**

The mechanisms that drives tolerance to tissue autoantigens is not fully understood as even APECED patients, who are genetically prone to develop autoantibodies, are tolerant against some common autoantigens. The hypothesis that some of the anti-cytokine antibodies commonly found in APECED patients may be protective should be investigated in larger series.

## Introduction

Autoimmune polyendocrinopathy candidiasis ectodermal dystrophy (APECED, OMIM 240300) or autoimmune polyendocrine syndrome type 1 (APS1) is a rare autosomal recessive disorder. It is caused by loss-of-function mutations in the autoimmune regulator (*AIRE*) gene, located on chromosome 21 (21q22.3) (1, 2, 3, 4). APECED predisposes early in life to chronic mucosal candidiasis and to the progressive development of various endocrine disorders, such as Addison's disease, primary hypoparathyroidism, primary hypogonadism, type 1 diabetes, hypothyroidism, and non-endocrine tissue-specific autoimmune disorders such as alopecia areata, vitiligo, autoimmune gastritis, pernicious anemia, hepatitis, enteropathy, nephritis, and keratitis (1, 2, 3, 4) with a strong impact on quality of life (5).

*AIRE* encodes for a 545 amino acid protein, whose domains are characteristic of transcriptional regulators and chromatin binding proteins (6). *AIRE* is involved in the induction of self-tolerance through the promiscuous expression of a high number of tissue-specific antigens on thymic medullary epithelial cells for presentation to maturing thymocytes (4, 5, 6, 7, 8, 9). Its loss of function leads to the escape of self-reactive T-cell from the thymus and autoimmunity (6, 8). APECED patients typically develop a variety of tissue-specific autoantibodies (10), which are predictive of the development of autoimmune diseases (3, 4) but also anti-cytokine antibodies such as type 1 interferons (IFN) and Th17-related interleukins 17 (IL17) and IL22 (11, 12). Interestingly, as AIRE is also expressed in peripheral dendritic cells and in the secondary lymphoid organs, AIRE may have relevance also in peripheral tolerance (13, 14). Despite an intra-familial and inter-individual variability, there is only a certain number of autoimmune diseases that APECED patients do develop and some common autoimmune diseases have not been reported, such as multiple sclerosis, bullous disorders, autoimmune thrombocytopenia or neutropenia, or Goodpasture syndrome. Cases of celiac disease, hemolytic anemia, and Sjögren's syndrome are anecdotal (2, 15), raising the question whether these associations are fortuitous or not.

The aim of this study was to assess the extent of additional circulating autoantibodies in a series of mainly adult Finnish APECED patients and their potential clinical relevance in case of detection. Autoantibodies for this study included antinuclear antibodies (AN-Abs); antibodies to extractable nuclear antigens (ENA-Abs, including smooth muscle (Sm-Ab), ribonucleoprotein (RNP-Ab), SSA/Ro-Ab, and SSB/La-Ab) for systemic lupus erythematosus, Sjögren's syndrome, and other connective tissue diseases; antibodies to the cyclic citrullinated peptide (CCP-Abs) for rheumatoid arthritis; antibodies to tissue transglutaminase (tTGM-Abs) for celiac disease; antibodies to the 180 kDa bullous pemphigoid antigen (BP180-Abs); and antibodies to desmoglein 1 (Dsg1-Abs) and Dsg3-Abs respectively. BP180-Abs are associated with BP while desmoglein antibodies with pemphigus vulgaris (Dsg3-Abs) and pemphigus foliaceus (Dsg1-Abs).

## Materials and methods

### Patients

Sera were collected prospectively from 2010 to 2012 from 30 Finnish APECED patients with confirmed mutations in *AIRE* gene. Sera from eight healthy blood donors were used as controls for each autoantigen, although the reference values of HUSLAB (http://www.huslab.fi), the largest university hospital laboratory in Finland, are based on the values in large normal population values as indicated in the accreditation documents of the laboratory (www.finas.fi). Because of limitations in the availability of some sera, AN-Abs, ENA-Abs, CCP-Abs, and TGA-Abs serology was performed on 24 patients while anti-epidermal antibodies in 30 patients. The clinical follow-up data of all patients as their diagnosis was available through their patient files and/or through a detailed, structured questionnaire and interview performed recently (5).

APECED was diagnosed at the mean age of 6 years (range, 0–19 years±4.9) among the recruited 30 patients (20 females and 10 males). At the time of the present serological analyses, their mean age was 38 years (range, 7–65 years±14.2) and the disease had evolved for 32 years (4.5–52 years±12.8). The main clinical manifestations of this APECED cohort are summarized in Table 1. The serological analysis was performed at one time point and in the same laboratory (HUSLAB) for all sera.

### Immunological assays

The following immunological assays were performed at the accredited Helsinki University Central Hospital laboratory, HUSLAB (http://www.huslab.fi) as follows. For AN-Abs, indirect immunofluorescent assays on HEp-2 cells were performed with NOVA Lite HEp-2 ANA Kits/Substrate Slides (NOVA Lite, San Diego, CA, USA) and with FITC-conjugated rabbit anti-human IgG as secondary antibody (Dako, Copenhagen, Denmark, Dako F0202). Identification of ENA-Abs included Sm-Abs, RNP-Abs, SSA/Ro, and SSB/La by fluorescence enzyme immunoassay. The cutoff values were defined as follows: 7 U/ml for SSA/Ro-Ab and SSB/La-Ab and 5 U/ml for RNP-Ab and Sm-Ab. CCP-Abs and tTGM-Abs were assessed by fluorescence enzyme immunoassay with EliA CCP (Thermo Scientific, Vantaa, Finland; 14-5515-01) and EliA Celikey IgA (Thermo Scientific, 14-5517-01) kits respectively with an ImmunoCap 250 Allergy ImmunoAssay Analyzer systems (Phadia AB, Uppsala, Sweden). IgA deficiency was screened simultaneously for celiac disease. The cutoff value for CCP-Abs was 7 U/ml. BP180-NC16a ELISA (specific for the immunodominant NC16a domain of the protein), with a cutoff value of 9 U/ml, was used for BP antibodies. Pemphigus vulgaris-related autoantigens were determined with an EIA assay for Dsg1 (MESACUP Desmoglein test ‘Dsg1’ ELISA, MBL Medical & Biological Laboratories Co., Ltd., Nagoya, Japan; cutoff value 14.0 U) and for Dsg3 (MESACUP Desmoglein test ‘Dsg3’ ELISA MBL Medical & Biological Laboratories Co., Ltd.; cutoff value 7.0 U).

The study was approved by the Medicine Ethical Review Board of Helsinki and Uusimaa Joint Authority (dnro 8/13/03/01/2009, research permission IAS09 APS1 6209/T101060071) and each participant signed an informed consent.

## Results

The patients displayed the typical clinical features related to APECED, namely past or active history of chronic candidiasis and various combinations of endocrine and non-endocrine disorders as previously reported for APECED (1, 2, 3, 4). None of the patients were currently under any high dose of corticosteroids or systemic immunosuppressive treatment that could have influenced the outcome of the immunological results here. Patients with Addison's disease had received replacement therapy with oral hydrocortisone at a daily dose of 15–25 mg since the diagnosis of the disease in their childhood.

The occurrence of the antibodies tested in this study is summarized in Table 2. AN-Abs were negative in 75% (18/24) of our patients. A weakly positive reaction (titer 1:80) was found in 6/24 patients (25%) with either a speckled (4/6) or a homogenous (2/6) pattern. The target antigen of the AN-Abs was positive for SSA/Ro in one patient at a borderline level (7 IU) who did not have any positivity for AN-Ab. None of the patients displayed a past or recent history suggestive of systemic lupus erythematosus, or other variant of cutaneous lupus, Sjögren's syndrome or other connective tissue diseases. We did not find any statistical difference regarding the prevalence of clinical manifestations and the presence or the absence of circulating AN-Abs at 1:80 titers (*χ* ^2^; Table 3).

CCP-Abs and tTGM-Abs were negative in all patients (24/24) and correspondingly none of the patients had been diagnosed with or presented with symptoms of rheumatoid arthritis or celiac disease (Table 2). Of note, no patient had IgA deficiency.

Two patients disclosed positive anti-BP180 antibodies with 20 and 31 U/ml (cutoff value 9 U/ml) respectively. Two other patients had weakly positive antibodies (8 and 10 U, cutoff value 7 U) against Dsg3, but not against Dsg1 (Table 2). None of the patients disclosed any symptoms or history indicating BP or pemphigus vulgaris. Of note, the two patients with circulating BP180 are aged 65 and 45 years.

In our control group, one out of eight healthy individuals had positive AN-Abs (1:320) without any specific target. The same individual disclosed a weak positivity for CCP-Abs (9 U/ml). This healthy individual did not have thus far any clinical diagnosis for systemic lupus or rheumatoid arthritis. The other controls did not display any positivity.

## Discussion

As *AIRE* is involved in the mechanisms of self-tolerance and APECED patients develop a wide number of autoimmune diseases, one would expect that APECED patients are prone to develop a larger variety of autoimmune diseases. This hypothesis prompted us to screen systematically our patients for autoantibodies associated with certain common autoimmune diseases, namely systemic lupus erythematous, rheumatoid arthritis, and other connective tissue diseases such as Sjögren's syndrome on one hand and celiac disease and bullous skin diseases (BP and pemphigus vulgaris) on the other hand. We chose to screen autoimmune diseases associated with an immunity against different classes of self-antigens according to their expression (16) such as i) self-antigens expressed constitutively in all cell types, ii) self-antigens of restricted tissue expression but present in the circulation at various levels, iii) self-antigens of restricted tissue expression that are undetectable in the circulation, and iv) sequestered antigens (16). To the best of knowledge, this is the largest series of APECED patients so studied. In our series, AN-Abs were found to be weakly positive (titer 1:80) in 25% of our patients and only one (4%) disclosed a very low positivity for SSA/Ro-Abs without any AN-Ab.

Our results differ from those of Obermayer-Straub *et al*. (17) who found a positivity of AN-Ab of 1:80 or over in 8.9–12.5% among a series of 64 Finnish APECED patients with or without autoimmune hepatitis. However, they used an indirect immunofluorescence method on frozen sections of rat liver and kidney tissue (17).

Perniola *et al*. (18) observed low titers of AN-Abs in their series of 11 Italian APECED patients and two of them had confirmed Sjögren's syndrome. However, they concluded that their patients did have ‘positive’ low titers of AN-Ab (9/10, 1:20–1:80). Besides, ENA-Ab was positive in 90% of their cases (9/10, SSA/Ro, SSB/La or RNP-Abs, 1:150). For our accredited AN-Ab method on the HEp-2 substrate, titers of 1:80 or higher are defined positive ((19), http://www.huslab.fi). However, at such titers, it is debatable to give any relevance in clinical practice to such ‘weakly’ positive in asymptomatic adults especially. As other authors stressed, low-titer ANAs (1:160 or less) may not need a further test for anti-ENA-Abs unless an ANA-associated disease is highly suspected (20). Of note, the only child of our series, aged 7 years, was negative for AN-Abs. In Finland, the prevalence of circulating AN-Abs in blood donors is >6% with a titer 1:80 and 3% with a titer 1:320 and thus such low titers are not considered informative for clinical diagnostics. A direct comparison between the two studies above is not possible but, when using the same cutoff value of 1:80, only 30% (3/10) of the patients in Perniola *et al*. (18) series display positivity, which is close to our results. However, a discrepancy between the ENA-Abs remains with 4% positive in our series vs 100% in the Italian series. Differences in the used techniques and commercial kits and different genetic background between our national cohort of APECED patients and the geographically isolated region of the South of Italy with a rare W78R *AIRE* mutation (21) may explain the differences in the results.

ENAs are nuclear/cytoplasmic ubiquitous molecules. As suggested by previous studies, it is possible that, because of the large availability of such self-antigens (even if promiscuous expression happens in medullary thymic epithelial cells), autoreactive T-cell clones are deleted at an early stage of development, probably during the double positive (CD4+CD8+) stage (22, 23). Such an AIRE-independent mechanism would eliminate autoimmunity against nuclear antigens also in APECED patients. However, B-cell differentiation takes place in the peripheral lymphoid tissue, and the organ-specific tissue destruction is likely to expose the patients to these antigens in the periphery.

BP is the most common autoimmune blistering skin disease, occurring most commonly in elderly patients (24). The clinical manifestations are related to autoantibodies directed against a 180 kDa protein, called the BP antigen 2 (BPAG2 or BP180 Ag) or type XVII collagen because of the presence of collagenous repeats in its extracellular domain. The autoantibodies bind to this antigen, a key component of the epidermal hemidesmosomes, and lead to a cascade of inflammatory events and finally to blister formation (24). In our APECED cohort, none of the patients disclosed symptoms suggestive of BP. However, two elder patients had low-level circulating BP180 antibodies. As circulating BP180 antibodies are predictive of relapses in patients with BP in clinical remission (25), only a long-term follow-up of our two patients will show whether the positivity of these antibodies is relevant or not. As the patients did not have any symptoms, not even itching, it was not feasible to perform direct immunofluorescence analysis of healthy skin to search for immunoglobulin deposits at the dermo-epidermal basal membrane junction. To the best of our knowledge, the role of AIRE in the expression of BP180 antigen has never been assessed. In the past, life expectancy of APECED patients was diminished for numerous reasons (acute adrenal crisis, sepsis, oral squamous cell carcinoma, depression, and suicide) making older age-associated autoimmune disorders unreported. Improvement of the patients' management and increased life expectancy may, however, reveal such autoimmune diseases in the future as BP occurs mainly in patients aged over 65 years (26). Besides, other factors occurring in the development of BP have to be taken into account (27).

The group of pemphigus diseases is characterized by blistering and ulceration of the skin, mucous membranes, loss of normal epithelial cell–cell adhesion (acantholysis), and the presence of autoantibodies directed against transmembrane desmosomal proteins (desmogleins). The pemphigus vulgaris antigen is a 130 kDa protein, Dsg3, which belongs to the cadherin superfamily of calcium-dependent adhesion proteins while Dsg1 is the target of autoantibodies in pemphigus foliaceus, a superficial variant of pemphigus (24). In our study, two patients presented anti-Dsg3-Abs without any clinical signs of pemphigus vulgaris. None had Dsg1-Abs. Interestingly, Wada *et al*. (28) recently studied the expression of Dsg3 in the thymus and found that Dsg3 expression in the thymic epithelial cells was *AIRE* dependent. However, they were unable to induce a pemphigus vulgaris phenotype in an *Aire* ^−/−^ mouse model (28) and the *Aire* ^−/−^ mouse does not develop any bullous skin lesion phenotype. This indicates that tolerance against this antigen is maintained despite *AIRE* deficiency. Our serological analysis confirms their observation and the intriguing contradiction between a self-antigen encoded by *AIRE* and the absence of putative consequences deriving from *AIRE* deficiency.

Celiac disease is one of the prototype of intestinal autoimmune disease and prevalent in Finland, diagnosed in 2.4% of the 30- to 64-year-old population (29). It is related to an adaptive immune response initiated by the interplay between MCH class 1 HLA-DQ2 and DQ8 (30). The question of other factors impairing immunoregulation is still unclear. However, rare cases of celiac disease have been reported among APECED patients. Betterle *et al*. (15) reported two cases in their series, which represent only 4% of their patients. We screened for IgA tTGM-Abs in our APECED cohort but failed to find any circulating tTGM-Abs, neither did any patient have celiac disease. As transglutaminase 2 is an ubiquitous soluble protein, its dependence to *AIRE* expression may be unlikely. However, APECED patients often present with an autoimmune enteropathy, directed against enterochromaffin cells, which can lead to severe malabsorption (31, 32). Therefore, we suggest screening for tTGM-Abs in case of chronic diarrhea, especially in countries with a high prevalence of celiac disease (32).

Finally, rheumatoid arthritis is one of the most common autoimmune diseases, affecting 0.5–1% of the population in Western countries. We did not choose rheumatoid factor (usually IgM isotype) as a screening antigen because of its low diagnostic specificity. CCP-Abs have a high specificity (91–98%) and are currently used for the diagnosis of RA. However, to improve sensitivities of the tests, cyclic synthetic peptides with a high content of citrulline are used as antigen (33), and although not a natural antigen, the absence of these antibodies excluded the presence of active rheumatoid arthritis or other inflammatory joint disorders in our patients. Interestingly, Campbell *et al*. (34) showed that the *Aire* ^−/−^ mouse developed a collagen-induced arthritis, an experimental model of rheumatoid arthritis. However, *Aire* ^−/−^ mouse does not spontaneously develop inflammatory arthritis.

The reason why APECED patients do not develop a comprehensive autoimmunity against ‘all’ peripheral tissue antigens is not fully understood. The simplest explanation for such rarity could be the rarity of APECED itself. As APECED is a rare disease, we may not have been able to detect the unusual autoimmune manifestations. Besides, as discussed earlier, autoreactive T-cell clones for ubiquitous molecules may be deleted at an early stage of development, preceding in this way AIRE-regulated events in the thymic medulla. Moreover, predisposition to the development of autoimmune diseases such as pemphigus or celiac disease is also related to HLA alleles (35, 36). The Finnish APECED patients do not differ from the normal population in their HLA allele distribution and APECED is not considered to disclose a specific HLA association (37). Betterle *et al*. (15) found in their series of patients an increased frequency of HLA DR3 and DR5, but no correlation with class 1 MHC. The APECED phenotype, as to the typical endocrine target organ disease, has been shown to be influenced by HLA class II (37, 38), but interestingly, the HLA alleles were not found to influence the autoantibody formation (37).

Lastly, an alternative hypothesis would be that APECED patients may develop not only ‘pathogenic’ autoantibodies but also ‘protective’ autoantibodies that would prevent development of some common autoimmune-type diseases. For example, high levels of IFNα and IFN-induced cytokines are typical for systemic lupus (39) and anti-IFN antibodies have shown therapeutic efficacy (40). Most of the present patients have been shown earlier to have high titers neutralizing autoantibodies against type 1 IFN and lower titer antibodies against type 3 IFN (12). Likewise, the anti-cytokine antibodies commonly found in APECED patients may be protective. However, the absence of disease development is difficult to prove in clinical patient series but would rather imply appropriate animal models to test the patient sera.

## Conclusion

Despite the loss of central tolerance, APECED patients do not present high titers of antibodies associated with connective tissue diseases such as systemic lupus erythematosus, Sjögren's syndrome, or rheumatoid arthritis. Celiac disease is most likely associated fortuitously with APECED. Lastly, there is to date no case of autoimmune bullous disease associated with APECED. The presence of autoantibodies against BP180 or Dsg3 is of undetermined significance thus far. Therefore, we find it unnecessary to perform a wide screening of autoantibodies in APECED patients on a routine basis but only in case of evocative clinical symptoms.

The mechanisms that drive tolerance to tissue autoantigens are not fully understood as even with *AIRE* deficiency and a proneness to develop autoantibodies, APECED patients do not seem to develop clinical autoimmunity indiscriminately.

## Author contribution statement

N Kluger participated in the design of the study, collected the data, analyzed the results, and drafted the manuscript. K Krohn and A Ranki participated in the design, analyzed the results, and coordinated the study. All authors contributed to and approved the final manuscript.

## Figures and Tables

**Table 1:** Disease components in the APECED patients of the present series

	**Prevalence**	
**Disease components**	*n*=30	%
Mucocutaneous candidiasis	28	93
Hypoparathyroidism	24	80
Addison's disease	24	80
Hypothyroidism	12	40
Hypogonadism	15	50
Female hypogonadism	13/20	65
Male hypogonadism	2/10	20
Alopecia/hair loss	14	46
Asplenia/hyposplenism	10	33
GH deficiency	8	26
Vitiligo	6	20
Diabetes mellitus type 1	5	16
Pernicious anemia	4	13
Autoimmune hepatitis	2	6

**Table 2:** Prevalence of autoantibodies in the APECED patients of the present series

**Type of autoantibody**	**Serology: positive/total cases**	**Comments**	**Clinical disease: present/total**
Antinuclear antibodies	6/24 (25%)	Titer 1:80; four speckled and two homogenous	Lupus erythematosus, autoimmune connective tissue disease: 0/24
Anti-extractable nuclear antigens	1/24 (4%): anti-Ro/SSA	Borderline value 7 U/ml (cutoff value 7 U/ml)	Lupus erythematosus, autoimmune connective tissue disease: 0/24
Anti-CCP	0/24		Rheumatoid arthritis: 0/24
Anti-transglutaminase	0/24		Celiac disease: 0/24
Anti-BP180	2/30	Low positivity: 20 and 31 U/ml, (cutoff value 9 U/ml)	Pemphigoid: 0/30
Anti-Dsg1	0/30		Pemphigus foliaceus: 0/30
Anti-Dsg3	2/30	Low positivity 8 and 10 U (cutoff value <7 U)	Pemphigus vulgaris: 0/30

**Table 3:** Prevalence of disease components of APECED according to the positivity of ANA (1:80)

**Disease components**	**ANA +1:80**	**ANA −1:80**	***P*<0.05** (*χ* ^2^ test)
	*n*=6 (3 F/3 M)	*n*=18 (12 F/6 M)	
Mucocutaneous candidiasis	6 (100%)	16 (88%)	NS
Hypoparathyroidism	5 (83%)	14 (77%)	NS
Addison's disease	5 (83%)	14 (77%)	NS
Hypothyroidism	4 (66%)	7 (38%)	NS
Hypogonadism	2 (33%)	10 (55%)	NS
Female hypogonadism	2 (2/3, 66%)	8 (8/12, 66%)	NS
Male hypogonadism	0	2 (33%)	NS
Alopecia/hair loss	2 (33%)	10 (55%)	NS
Asplenia/hyposplenism	3 (50%)	5 (28%)	NS
GH deficiency	1 (16%)	6 (33%)	NS
Vitiligo	1 (16%)	4 (22%)	NS
Diabetes mellitus type 1	2 (33%)	2 (11%)	NS
Pernicious anemia	1 (16%)	2 (11%)	NS
Autoimmune hepatitis	0	2 (11%)	NS

F, female; M, male; NS, non-significant.
